# Efficient Implementation of a Symbol Timing Estimator for Broadband PLC

**DOI:** 10.3390/s150820825

**Published:** 2015-08-21

**Authors:** Francisco Nombela, Enrique García, Raúl Mateos, Álvaro Hernández

**Affiliations:** Electronics Department, University of Alcalá, Campus Universitario s/n, Alcalá de Henares, Madrid 28805, Spain; E-Mails: francisco.nombela@depeca.uah.es (F.N.); enrique.garcia@depeca.uah.es (E.G.); raul@depeca.uah.es (R.M.)

**Keywords:** Power-Line Communications, symbol timing estimation, complementary sequences, Zadoff-Chu sequences, FPGA-based architecture

## Abstract

Broadband Power Line Communications (PLC) have taken advantage of the research advances in multi-carrier modulations to mitigate frequency selective fading, and their adoption opens up a myriad of applications in the field of sensory and automation systems, multimedia connectivity or smart spaces. Nonetheless, the use of these multi-carrier modulations, such as Wavelet-OFDM, requires a highly accurate symbol timing estimation for reliably recovering of transmitted data. Furthermore, the PLC channel presents some particularities that prevent the direct use of previous synchronization algorithms proposed in wireless communication systems. Therefore more research effort should be involved in the design and implementation of novel and robust synchronization algorithms for PLC, thus enabling real-time synchronization. This paper proposes a symbol timing estimator for broadband PLC based on cross-correlation with multilevel complementary sequences or Zadoff-Chu sequences and its efficient implementation in a FPGA; the obtained results show a 90% of success rate in symbol timing estimation for a certain PLC channel model and a reduced resource consumption for its implementation in a Xilinx Kyntex FPGA.

## 1. Introduction

In recent years Power-Line Communications (PLC) have emerged as a consolidated broadband standard for data transmission [1], taking advantage of the mains already installed in most indoor environments (public buildings, homes, industrial factories, *etc.*). Apart from facilitating the deployment of devices since there is no need for additional cabling, this approach provides feasible solutions to different kinds of applications, such as sensory and automation systems [2,3,4], distributed systems [4,5], smart spaces [6,7], or even industrial networks [8,9], where it is necessary to have available a broadband communication among the elements that integrate the system.

Nevertheless, PLC-based systems still have some details that can be improved in order to achieve a better performance. One of them is the implemented medium access technique, where most of the previous works have been focused on Multi-Carrier Modulation (MCM) [10,11], not only in PLC but also in other standards [12] like Long Term Evolution (LTE) [13]. MCM allows the performance and the spectrum efficiency to be enhanced by dividing the available bandwidth into subchannels, which data are transmitted through. Different MCM techniques have been successfully proposed for PLC, depending on how the subchannel division is carried out. Some of the most relevant are those based on Discrete Trigonometric Transform (DTT) [14], or those based on filter banks (Filter-Bank Multi-Carrier, FBMC) [10,15]. In any case, no matter the considered medium access technique, all the approaches require a suitable and reliable synchronization method between transmitters and receivers, in order to be capable of achieving the expected performance, thus avoiding inter-symbol interference (ISI) and inter-carrier interference (ICI).

The synchronization issue has been widely considered in previous works for multi-carrier techniques, as Discrete Modulation Tone (DMT) [16], FBMC [17], or Orthogonal Frequency Division Multiplexing (OFDM) [18,19] for wireless communications.

Nevertheless, for broadband PLC there is a reduced number of works that deal with synchronization in such a scenario, where some features from the PLC channel, such as selective frequency fading, channel length, and noise models should be considered [20]. Most of the research done for PLC is focused on the OFDM implementation proposed in the IEEE 1901–2010 standard [21], but not on the FBMC physical layer approach. In those works, the auto-correlation metric is typically used, which consists on transmitting several repeated symbols and correlating the consecutive symbols in the receiver [18,22]. This metric is useful for wireless communications as it increases the robustness to Doppler shifts, but it suffers from the specific conditions of PLC channel. Furthermore, previous PLC synchronization works consider the maximum peak of the auto-correlation metric as the beginning of a symbol [23,24]. This cannot be assumed in practice due to multipath and therefore the first arriving path can be of lower magnitude than a multipath component [25]. This is the reason why extra research efforts should be focused on the proposal and development of suitable synchronization algorithms for MCM in PLC communications.

Generally speaking, it is relevant to note that many previous proposals, not only for synchronization but also for multi-carrier medium access techniques, imply a challenge from a real-time implementation and sensory point of view [26,27]. They often handle high data rates, requiring intensive and parallel signal processing and a certain connection to digital converters and sensors. Accordingly, the possibility of providing a feasible real-time architecture for the implementation of the proposed synchronization algorithm actually becomes significant. Recent Field-Programmable Gate Array (FPGA) devices already play a key role in the implementation of this kind of systems [28]. They allow the design of highly parallel and flexible architectures for signal processing at high frequencies (in the range of MHz).

This work presents a novel algorithm to estimate the synchronization delay, suitable for a FBMC transmultiplexer used in PLC communications. The proposed synchronization is based on cross correlation, where the received signal is correlated with the transmitted one. Unlike the common approaches based on auto-correlation techniques, the proposal makes possible to improve performance and robustness of communications in the PLC channel. Furthermore, a FPGA-based architecture is also described for the real-time implementation of the proposed algorithm, optimizing terms as resource consumption and operation frequencies, and increasing resource reutilization. The rest of the manuscript is organized as follows: Section 2 reviews the medium access technique considered for PLC synchronization, whereas Section 3 explains the proposed synchronization algorithm; Section 4 describes the hardware architecture proposed for the implementation of the synchronization algorithm; Section 5 shows some experimental results that validate the design; and, finally, conclusions are discussed in Section 5.

## 2. Wavelet-OFDM Approach

Wavelet-OFDM is a medium access technique that can be efficiently implemented by means of the Discrete Cosine Transform (DCT), also known as a technique based on Cosine-Modulated Filter Bank (CMFB). According to the IEEE 1901–2010 standard [21], in narrowband communications through mains, Wavelet-OFDM modulation is considered robust against selective frequency fading and narrowband noise, thus providing a better use of the available bandwidth since no guard intervals are required, unlike OFDM. Furthermore, the IEEE 1901–2010 standard defines frequencies at which PLC systems can transmit. For that purpose, *M* = 512 carriers are distributed in the baseband version, for the range from 0 to 31.25 MHz, although only those in the range 1.8–28 MHz can be actually used. Even in this range, a mask has to be applied to filter frequencies related to amateur radio, so, finally, a set of 360 carriers is available for information transmission.

**Figure 1:**
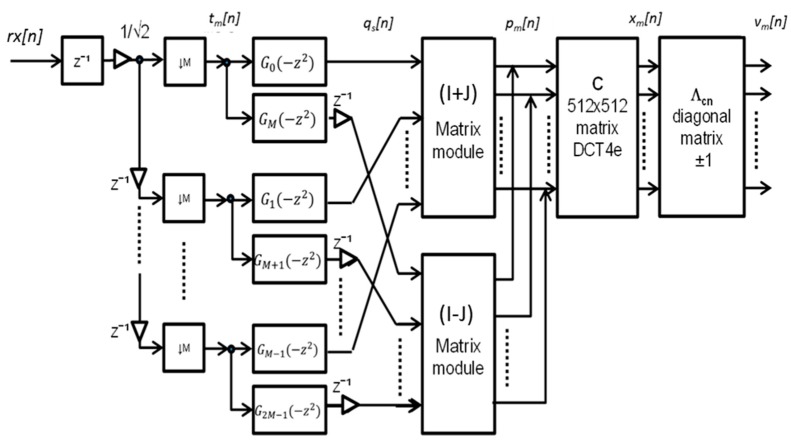
General block diagram of the used Wavelet-OFDM receiver.

Figure 1 shows the block diagram of a possible efficient implementation of the filter bank at the Wavelet-OFDM receiver [28], based on the DCT-4e. Basically, the processing consists of a first deserialization of the received signal *rx*[n] to obtain the *M* different subchannels *t_m_*[n]. These *M* subchannels are processed by pairs of filters *G_s_*(*z*^−1^), where *s* = 0, 1, …, *S*−1 with *S* = 2·*M*, to obtain the intermediate signals *q_s_*[n]. Afterwards, these signals *q_s_*[n] are linearly operated by matrices (**I** + **J**) and (**I** − **J**) and added, so the DCT-input signals *p_m_*[n] are obtained. The DCT module processes the *M* subchannels to provide the signals *x_m_*[n], which, after multiplied by the diagonal matrix **Λ_cn_**, provide the output subchannels *v_m_*[n].

This efficient implementation can be expressed in a matricial way for the synthesis bank **f**(*z*) as Equation (2):

(1) $$f\left(z\right)={\left[F_{0}\left(z\right)\;F_{1}\left(z\right)\;\ldots F_{M-1}\left(z\right)\right]}^{T}$$

(2) $$f\left(z\right)=\frac{1}{\sqrt{2}}\cdot \left[\begin{array}{cc} g_{o}\left(z^{2M}\right) & z^{-M}g_{1}\left(z^{2M}\right) \end{array}\right]\cdot \left[\begin{array}{c} \left(I+J\right)\\ \left(I-J\right) \end{array}\right]\cdot C_{4e}\cdot \Lambda_{cn}$$

And for the analysis bank **h**(*z*) = **f**(*z*^−1^) as Equation (3):

(3) $$h\left(z\right)=\frac{1}{\sqrt{2}}\cdot \Lambda_{cn}\cdot C_{4e}\cdot \left[\begin{array}{cc} \left(I+J\right) & \left(I-J\right) \end{array}\right]\cdot \left[\begin{array}{c} g_{o}\left(z^{2M}\right)\\ \;z^{-M}g_{1}\left(z^{2M}\right) \end{array}\right]$$

where **g_0_**(*z*) is a diagonal matrix, whose diagonal elements are [*G*_0_(–*z*), *G*_1_(–*z*),…, *G_M_*_–1_(–*z*)] and **g_1_**(*z*) is also a diagonal matrix whose diagonal elements are [*G_M_*(–*z*), *G_M+_*_1_(–*z*),…, *G*_2*M–1*_(–*z*)], where *G_M_*(–*z*), 0 ≤ *m* ≤ 2·*M* – 1, is the prototype filter in the discrete domain; **C_4e_** is the DCT-4e matrix, whose elements are:

(4) $${\left[C_{4e}\right]}_{k,l}=\sqrt{\frac{2}{M}}\cdot \cos\left(\left(k+\frac{1}{2}\right)\frac{\text{π}}{M}\cdot \left(l+\frac{1}{2}\right)\right),\;0\leq k\leq M-1,\;0\leq l\leq 2M-1$$

**Λ_cn_** is a *M* × *M* diagonal matrix, whose *i*-th element is:

(5) $${\left[\Lambda_{cn}\right]}_{i,i}=\sqrt{2}\cdot \cos\left(\frac{\pi }{2}\left(i+\frac{1}{2}\right)\right)\cdot \cos\left(\theta_{c}\right),\;\theta_{c}=\left\{0,\text{π}\right\}$$

and **I** denotes a *M* × *M* identity matrix, whereas **J** denotes the counter-identity matrix.

In the case of a bank with perfect reconstruction, then *R*(*z*)·**f**(*z*) = **h**(*z*)·*R*(*z*), where *R*(*z*) is the input to the synthesis bank. Nonetheless, ideal synchronization estimation is assumed to achieve a perfect reconstruction filter bank, otherwise the reconstructed signal is affected by ISI and ICI, making unfeasible to recover the transmitted data at the receiver side. For that reason it is significant to obtain the design and implementation of an efficient synchronization algorithm, able to accurately estimate the symbol timing.

## 3. Proposed Synchronization Algorithm

### 3.1. Multi-Level Complementary Sequences and Zadoff-Chu Sequences

The synchronization algorithm proposed here for a Wavelet-OFDM receiver is based on the use of multi-level complementary sequences or Zadoff-Chu sequences as pilot signals, since they provide suitable correlation properties and a flexible length, which allow the proposal to be adapted to the number of available subcarriers for transmission. Both types of sequences have been already used in numerous sensory systems described in previous works due to these correlation properties [29,30].

Multi-level complementary sequences present ideal correlation properties, so that a set of *K* sequences *s_j_*_,*i*_, 0 ≤ *j* and *i* ≤ *K*−1 of length *L* is a complementary set of sequences (CSS) if the sum of their auto-correlation functions *Cs_j_*_,*i*_ is a Kronecker delta [31], according to Equation (6):

(6) $$\sum_{i=0}^{K-1}C_{s_{j,i}}\left[n\right]=\eta \cdot \text{δ}\left[n\right];\;0\leq j\leq K-1;\;\eta \in \mathbb{R}-\left\{0\right\}$$

Two CSS are not correlated if the sum of the aperiodic cross-correlation functions $C_{s_{j,i}s_{j^{\prime },i^{\prime }}}$, between the sequences *s_j,i_* and *s_j’_*_,*i’*_ from both sets, is zero for any correlation shifting Equation (7):

(7) $$\sum_{i=0}^{K-1}C_{s_{j,i}s_{j^{\prime },i^{\prime }}}\left[n\right]=0;\;0\leq n\leq L-1;\;0\leq j\neq j^{\prime }\leq K-1$$

where *K* is the maximum number of uncorrelated CSS and equal to the number of sequences in any set*.* In a similar way to complementary sequences, Zadoff-Chu sequences present other properties that become relevant when performing cross-correlations. The Zadoff-Chu sequences are non-binary codes with a constant module. They have a null correlation function between a certain Zadoff-Chu sequence *s_q_*[n] and a circularly shifted version of it, *s_q_*[*n* + Δ], except when they are aligned (Δ = 0), where *L* is the length of the mentioned sequence *s_q_* and 0 ≤ *q* ≤ *L* − 1 is the number of spreading sequences with low cross-correlation values according to Equation (8):

(8) $$\sum_{n=0}^{L-1}s_{q}\left[n\right]s_{q}^{*}\left[n+∆\right]=\{\begin{array}{c} 1\;∆=0\\ 0\;∆\neq 0 \end{array}$$

where $s_{q}^{*}$ is the complex conjugate of $s_{q}$ . This property allows a correct estimation of the temporal synchronization, as well as the estimation of the channel impulse response.

By selecting a prime number for the length *L*, the number of sequences that provide a minimum value in the cross correlation is equal to *L* − 1 [32]. Furthermore, if a non-prime length is required, it is possible to generate another sequence by means of truncation or cyclic expansion.

Finally, other property to be remarked is that the Discrete Fourier Transform (DFT) of a Zadoff-Chu sequence *s_q_* is another cyclically shifted sequence, thus implying that they can be generated both in time and frequency domains. This feature is relevant since processing often requires working in the frequency domain, so the correlation properties of these sequences are not lost in those cases.

### 3.2. Description of the Algorithm

**Figure 2:**
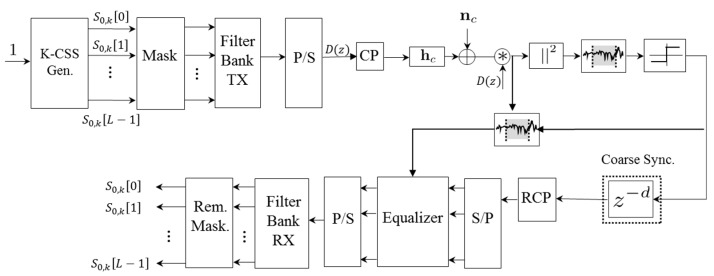
General block diagram of the signal processing modules involved in the PLC link.

Figure 2 shows a block diagram of the proposed synchronization algorithm, as well as the channel estimation and equalization modules. The proposed pilot-based symbol timing estimation uses cross-correlation techniques for PLC channel as it provides a better performance than the auto-correlation metric [33,34], and due to the negligible Doppler effect in PLC channels.

The ideal correlation features provided by multi-level complementary sequences and Zadoff-Chu sequences allow the improved detection of the first arrival tap, in order to develop a more robust synchronization algorithm. For representation purposes, a CSS generator of length *L* has been considered in Figure 2 and shown as *K-CSS Gen*. Nonetheless, Zadoff-Chu sequences can also be used for synchronization purposes.

Given a set of multi-level complementary sequences in the Z-domain:

$$S_{0}\left(z\right)=\left\{S_{0,0}\left(z\right),S_{0,1}\left(z\right),\ldots ,S_{0,K-1}\left(z\right)\right\}$$

where:

$$S_{0,k}\left(z\right)=s_{0,k}\left[0\right]+s_{0,k}\left[1\right]\cdot z^{-1}+\ldots +s_{0,k}\left[L-1\right]\cdot z^{-L+1}$$

the transmission of a set of *K* symbols with *M* samples is proposed, each one formed by a multi-level complementary sequence $S_{0,k}\left(z\right)$ of length *L* (*L* ≤ *M* and equal to the number of available subchannels). This number is determined by a transmission mask defined in the IEEE 1901–2010 standard [21]. The sequence bit $s_{0,k}\left[n\right]$ is assigned to the k-th symbol and to the n-th subchannel available in the synthesis bank, 0 ≤ n ≤ *L* − 1. In those subchannels that are not available for transmission due to PLC standard regulation, the bit 0 is assigned, so *M* − *L* subchannels will not be used.

In this paper we use a *K* = 2 multilevel CSS as a pilot waveform, thus transmitting 2 symbols (2·*M* samples) for synchronization purposes. On the other hand, in the case of the complex-valued Zadoff-Chu sequences, the bits are allocated in a similar way to the multi-level CSS, but the first symbol of *M* samples to be sent contains the real part of the sequence, whereas the second symbol contains the imaginary one.

**Figure 3:**
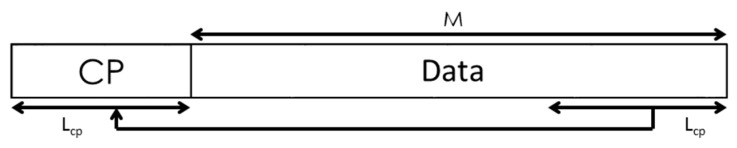
Block diagram of the CP insertion with length *L_cp_* in a symbol of length *M*.

The input sequence bits, after being allocated in the appropriate subcarrier by the mask block, are processed in parallel by the synthesis bank **f**(*z*) (*Filter Bank TX* in Figure 2), to shape the spectrum as designed in the prototype filters *G_m_*(−*z*). Later the output from **f**(*z*) is converted into a serial datastream by using a parallel-to-serial converter (*P/S* in Figure 2). Consequently, the output signal, after the *P/S* block is equal to Equation (9):

(9) $$D\left(z\right)=\sum_{k=0}^{K-1}\sum_{\begin{array}{c} n=0\\ n\in \chi \end{array}}^{L-1}s_{0,k}\left[n\right]\cdot F_{\chi }\left(z^{M}\right)\cdot z^{-\left(kM+\text{χ}\right)}$$

where χ is the subset of available subcarriers. After the appropriate codes have been used as a pilot signal, the last samples of the modulated symbol are repeated at the beginning of it, as shown in Figure 3. This constitutes the so-called Cyclic Prefix (*CP* in Figure 2), which allows ISI and ICI to be efficiently mitigated in the frequency domain when the channel delay spread is shorter than the CP length *L_cp_*. Although it is theoretically not necessary to use CP to avoid ISI and ICI in Wavelet-OFDM, its use efficiently reduces them by using a frequency domain equalizer with one complex multiplier per subcarrier [35].

Assuming no CP is used and there is an ideal channel (**h**_c_ = 1 in Figure 2) and no channel noise (**n**_c_ = 0), the correlation of the transmitted pilot at the receiver input is equal to:

(10) $$D\left(z\right)\cdot D\left(z^{-1}\right)=\sum_{k=0}^{K-1}\sum_{\begin{array}{c} n=0\\ n\in \chi \end{array}}^{L-1}s_{0,k}\left[n\right]\cdot F_{\text{χ}}\left(z^{M}\right)\cdot z^{-\left(kM+\text{χ}\right)}\cdot \sum_{k=0}^{K-1}\sum_{\begin{array}{c} n=0\\ n\in \chi \end{array}}^{L-1}s_{0,k}\left[n\right]\cdot F_{\text{χ}}\left(z^{-M}\right)\;\cdot z^{\left(kM+\chi \right)}$$

Assuming a good filter design, the correlation of adjacent subcarriers can be neglected, reducing the previous expression Equation (10) to Equation (11), where only one side of the correlation function is considered:

(11) $$D\left(z\right)\cdot D\left(z^{-1}\right)=\sum_{k=0}^{K-1}\sum_{\begin{array}{c} n=0\\ n\in \chi \end{array}}^{L-1}{\left(s_{0,k}\left[n\right]\right)}^{2}\cdot F_{\text{χ}}\left(z^{M}\right)\cdot F_{\text{χ}}\left(z^{-M}\right)+\sum_{k=0}^{K-1}\sum_{k\prime =k}^{K-1}\sum_{\begin{array}{c} n=0\\ n\in \chi \end{array}}^{L-2}s_{0,k}\left[n\right]\cdot s_{0,k\prime }\left[n+1\right]\cdot F_{\text{χ}}(z^{M})\cdot F_{\text{χ}+1}(z^{-M})\cdot z^{-M(k\prime -k)-1}$$

The first term in Equation (11) corresponds to the auto-correlation of the spreading sequences, affected by the auto-correlation of the filter bank, and the second term represents the cross-correlation of adjacent subcarrier filters and sequence bits.

In the case of having a real PLC channel and different noises affecting the channel [20], Equation (11) turns out a cross-correlation between the received signal *R*(*z*) and *D*(*z*), where *R*(*z*) = *D*(*z*)·**h**_c_ + **n**_c_ and expressed as Equation (12):

(12) $$R\left(z\right)\cdot D\left(z^{-1}\right)=h_{c}\sum_{k=0}^{K-1}\sum_{\begin{array}{c} n=0\\ n\in \chi \end{array}}^{L-1}{\left(s_{0,k}\left[n\right]\right)}^{2}\cdot F_{\text{χ}}\left(z^{M}\right)\cdot F_{\text{χ}}\left(z^{-M}\right)+D(z^{-1})\cdot n_{c}+h_{c}\cdot \sum_{k=0}^{K-1}\sum_{k\prime =k}^{K-1}\sum_{\begin{array}{c} n=0\\ n\in \chi \end{array}}^{L-2}s_{0,k}\left[n\right]\cdot s_{0,k\prime }\left[n+1\right]\cdot F_{\text{χ}}(z^{M})\cdot F_{\text{χ}+1}(z^{-M})\cdot z^{-M(k\prime -k)-1}$$

Therefore, the ideal correlation properties of multilevel complementary sequences are degraded by the correlation of the subcarrier filters, which is only ideal when all the subcarriers of the filter bank are used for transmission, and by the channel noise **n**_c_. Also, note that the CP insertion does not degrade the correlation properties of the spreading sequences. The same applies when using Zadoff-Chu sequences.

**Figure 4:**
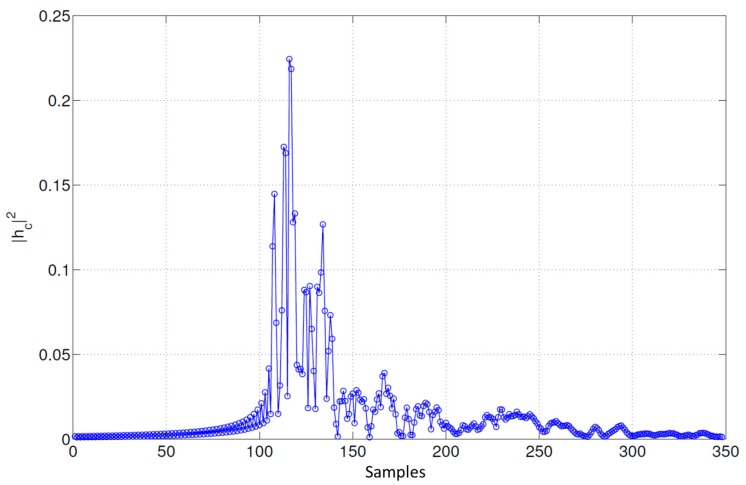
Module |*h_c_*|^2^ of the Tonello PLC channel impulse response.

After the CP insertion, the signal goes through the PLC channel **h**_c_ and the channel noise **n**_c_, as depicted in Figure 2. Hereinafter, the Tonello PLC channel model proposed in [36] has been considered for simulation, as well as the three different types of noises modelled in [20] for PLC. The Tonello channel model shows the harsh conditions of the PLC channel as the first tap does not necessarily present the highest amplitude, being challenging to estimate the first arriving path. Figure 4 shows the module |*h_c_*|^2^ of a realization of the Tonello PLC channel model sampled at 62.5 MHz, where the strong multipath and long channel duration can be observed.

In order to estimate the first arriving path, the cross-correlation derived in Equation (12) is squared and the maximum correlation peak is used to apply a window of 40 samples; within this window, the first sample higher than a certain static threshold is considered as the first tap of the channel impulse response.

After estimating and compensating the synchronization delay, *Coarse Sync* in Figure 2, the CP is removed at the receiver (*RCP* block in Figure 2). Note that if the symbol timing estimation error is lower than the CP length *L_cp_*, this error appears as a phase rotation for a frequency-domain equalizer, which can be efficiently compensated by using only one complex multiplier per subcarrier. For that purpose, the windowed correlation can be reused for channel estimation, thus minimizing the required computational load. Finally, the equalized signal is demodulated by using the analysis filter bank (*Filter Bank RX* in Figure 2), recovering the transmitted baseband symbols after removing the emission mask (*Rem. Mask* block in Figure 2).

## 4. Proposed Architecture

Some previous works have already dealt with the implementation of the Wavelet-OFDM emitter and receiver [28], even describing in detail the efficient architecture proposed for the filter banks. Because of that, this section is focused on the definition and design of an efficient architecture for the implementation of the proposed synchronization algorithm. Furthermore, it is important to remark that the proposal has been developed on a Xilinx XC7K325T FPGA, whose internal architecture, datawidth and resource availability determine certain design decisions, especially those related to the fixed-point representation of the involved signals. This fixed-point representation has been defined hereinafter by a format Q(α·β), where α is the global number of bits and β is the number of fractional bits.

**Figure 5:**
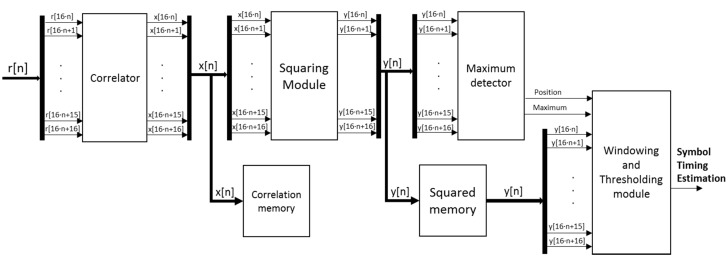
General block diagram of the architecture proposed for the implementation of the synchronization algorithm.

Figure 5 shows the block diagram of the synchronization proposal, according to the algorithm description carried out before. This diagram can be divided into four main modules: a correlator, a squaring module, a maximum detector, and a windowing and thresholding module. The global input for the synchronization architecture are the 2·*M* received samples *r*[n], obtained after discarding the cyclic prefix CP, whereas the final output is the estimated synchronization delay. Note that the input at the correlator has been parallelized in sets of 16 samples. This parallelism degree has been fixed in order not to significantly increase the resource consumption (especially multipliers), and, although it implies a latency in the global system operation, it is still possible to achieve real-time performance.

The critical issue about the design is to achieve real-time operation without discarding data at the reception, so the system should estimate the synchronization delay as fast as possible. Keeping this in mind, the most significant bottleneck is the correlation module, where a larger number of clock cycles are required to compute the corresponding correlation value *x*[n]. The approach based on computing the correlation function *x*[n] by means of sliding windows over the input data *r*[n] has been rejected [27], since the computational load becomes unfeasible for a real-time implementation. On the other hand, an approach based on non-overlapped windows over the input data *r*[n] has been considered here. At this moment, it is important to recall the importance of the cyclic prefix to perform this kind of correlation, in order not to discard a part of the information and degrade the correlation maximum value *x*[n], so only one block of 2·*M* samples, *i.e.*, two symbols of *r*[n], is processed to estimate the symbol timing.

**Figure 6:**
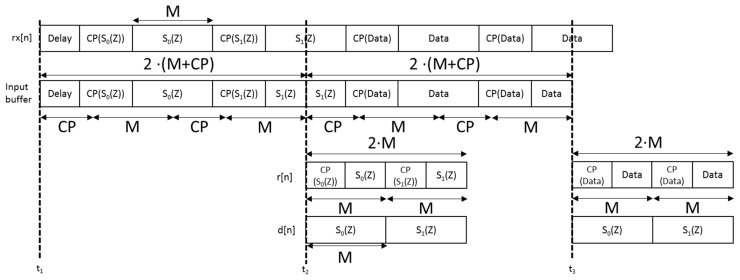
Scheme of the CP removal process, and the corresponding input for the correlation module.

The correlation module between the signal *r*[n] and the transmitted preamble *d*[n] involves the FFT and IFFT modules, which require most of the multipliers available in the FPGA. The proposed correlation module is based on the continuous acquisition at the receiver, signal *rx*[n], and the storing in an input buffer with a length of 2(*M* + *L_cp_*), where *M* is the length of every packet and *L_cp_* the cyclic prefix length (see Figure 6). Afterwards, it is possible to discard the cyclic prefix and obtain the input of the correlation module *r*[n] with length 2·*M* samples. Note that the transmitted preamble *d*[n] consists of, either a pair of multi-level CSS, each one allocated in a different packet, or a Zadoff-Chu sequence. In this last case, the real and the imaginary parts are transmitted separately, so two data packets are still required for the preamble *d*[n].

After removing the CP, the obtained signal *r*[*n*] should be correlated with the preamble *d*[*n*] modulated by the synthesis bank. It is assumed a constant preamble, and it only changes whether a different sequence is transmitted. This correlation can be expressed in the frequency domain as Equation (13):

(13) x[n]=IDFT{DFT{r[n]}·DFT{d[n]}}

where *x*[*n*] is the correlation output; DTF and IDFT mean the direct and inverse Discrete Fourier Transform, respectively; *r*[*n*] is the input buffer after CP removal; and *d*[*n*] is the transmitted preamble, both with a length of 2 *M*.

The fact of implementing both the DFT and the IDFT significantly increases the multiplier consumption in the design. Taking into account the DFT properties, it can be concluded that it is not necessary to implement an IDFT, since it is possible to reutilize the DFT to obtain the time-domain correlation. Simplifying Equation (13):

(14) X[k]=DFT{r[n]}·DFT{d[n]}=R[k]·D[k]

Since the IDFT is similar to DFT, with different sign in the exponential factor and different scaling constant, the correlation process can be rewritten as Equation (15):

(15) $$x\left[n\right]=IDFT\left\{X\left[k\right]\right\}=\frac{1}{N}\cdot {\left(DFT\left\{X^{*}\left[k\right]\right\}\right)}^{*}$$

where *N* is the length of the signal to be correlated; and *X*[*k*]* is the complex conjugate of *X*[*k*]. If it is assumed that the signals *r*[*n*] and *d*[*n*] are real-valued, it is possible to discard the imaginary part at the correlation output *x*[*n*], so:

(16) $$x\left[n\right]=\frac{1}{N}\cdot {\left(DFT\left\{X^{*}\left[k\right]\right\}\right)}^{*}=\frac{1}{N}\cdot \left(DFT\left\{X^{*}\left[k\right]\right\}\right)$$

Taking into account Equation (14), it can be obtained Equation (17):

(17) $$x\left[n\right]=DFT\left\{\frac{1}{N}\cdot {\left(R\left[k\right]\cdot D\left[k\right]\right)}^{*}\right\}$$

**Figure 7:**
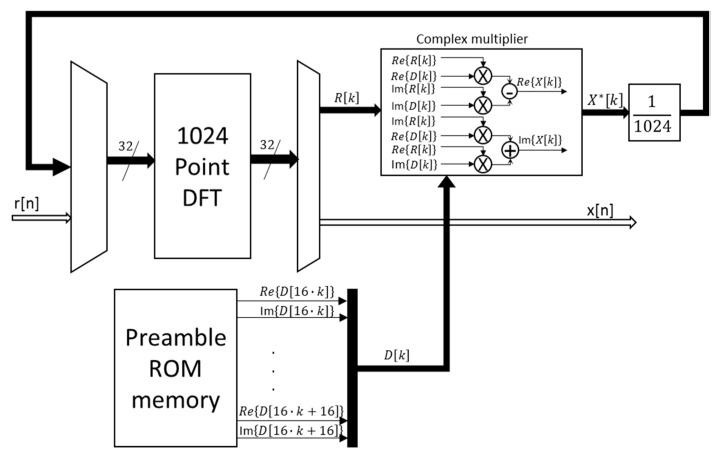
General block diagram of the architecture proposed for the implementation of the correlation module.

Figure 7 shows the block diagram proposed for the implementation of the correlation module, according to the optimizations described previously. It can be observed how the 1024-point FFT is reutilized to compute the time-domain correlation.

At the first stage, the system carries out the DFT for the input signal *r*[*n*] with a length of 2·*M* samples. The DFT block has 32 parallel inputs, half for the real part of the input samples and the other half for the imaginary part. Since the received signal *r*[*n*] is real, the imaginary inputs are null at this first stage. Not only the input data but also the outputs are represented in fixed point, with a format Q(18.8).

At the second stage, the complex multiplication between the output data from the DFT of the input signal *r*[*n*] and the output data from the DFT of the preamble *d*[*n*] is carried out. As has been already mentioned before, since the preamble is constant, its DFT can be computed off-line and stored in the corresponding Preamble ROM memory shown in Figure 7. The available multiplication cells, DSP48E1, are 25 × 18 bits, so, in order to use all the available data span, the stored DFT data for the preamble *d*[*n*] have a format Q(25.15). The output of the multipliers is divided by a scaling factor 1/*N* and truncated to the DFT format Q(18.8). Finally, in the last stage, the DFT block is involved again to compute the time-domain correlation, thus providing the output signal *x*[*n*] in the format Q(18.8) as well.

The correlation memory block in Figure 5 has been included to store the resulting correlation *x*[*n*], so it can be used later in the channel estimation and the computation of the equalizer coefficients. When the correlation module starts to provide valid output data after an initial latency, it is capable of generating 16 18-bit samples *x*[*n*] every clock cycle until the final length of 1024 samples. The squaring module consists of 16 multipliers that compute in parallel the squaring of signal *x*[*n*] in a format Q(36.16). This squared signal *y*[*n*] is truncated to the most significant 18 bits, Q(18.0), since the higher a correlation maximum is, the easier its detection becomes. The squared correlation function *y*[*n*] is also stored in a squared memory block to be used in the windowing and thresholding module, whereas the squared values *y*[*n*] are also processed by the maximum detector to determine the exact position of the correlation peak. Both the correlation and the squared memory blocks have a size of 64 × 288 bits, corresponding to a set of 16 samples with 18 bits each. This makes possible to read/write a whole set of 16 samples in a single memory access.

**Figure 8:**
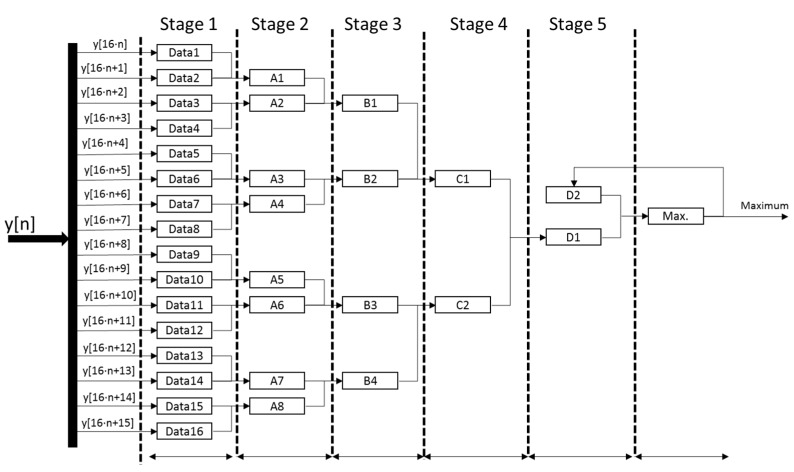
General scheme proposed for the implementation of the detector of the maximum correlation values.

The detection of the maximum correlation value consists of a successive and pipelined comparison to determine the maximum value among every set of 16 samples *y*[*n*] coming from the squaring module. Every clock cycle, a new set of 16 samples *y*[*n*] is inserted, and, by comparing in pairs at every clock cycle, the local maximum is obtained after four clock cycles. A last clock cycle is dedicated to determine the global maximum for the global length of 1024 samples, by comparing the local maximum values from every set of 16 samples. Figure 8 shows the scheme for the implementation of this maximum detector. Since the correlation length is 1024 samples and 16 new samples are inserted every clock cycle, 64 clock cycles plus a latency of five cycles are required to obtain the maximum correlation value and determine its position in the squared memory block.

Finally, the windowing and thresholding module is in charge of searching the first sample *y*[*n*] over a certain threshold before the detected maximum value, since that sample *y*[*n*] is considered as the start of the PLC channel. The windowing is implemented by reading the squared memory block at the position where the detected correlation peak is stored. Since every memory access provides 16 samples *y*[*n*] and three reading accesses are carried out, the length of the final window varies from 32 to 47 samples, depending on the exact position of the detected maximum value in the set of 16 samples *y*[*n*].

**Figure 9:**
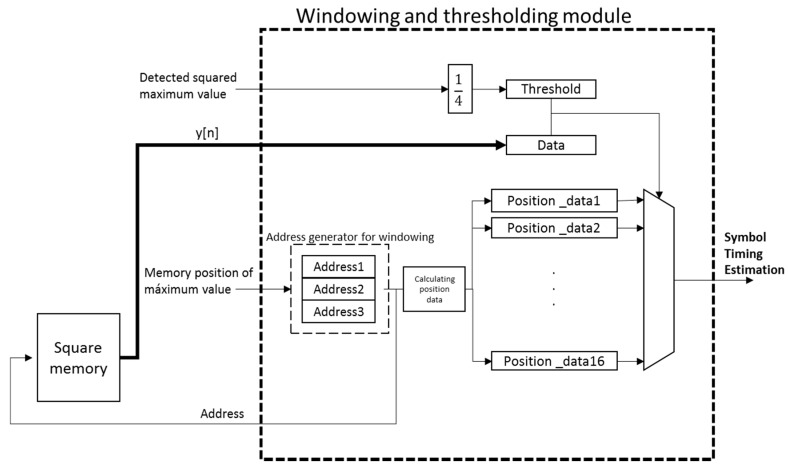
Block diagram of the proposed windowing and thresholding module.

The start of the PLC channel is searched in this window, and it is defined by the first value over a certain threshold. This threshold is experimentally fixed at the 25% of the detected maximum squared value *y*[*n*] and it should be updated for every PLC channel condition to effectively estimate the first arriving path. Figure 9 shows the block diagram of the windowing and thresholding module, where the input signals are the detected maximum squared value *y*[*n*] and its corresponding location in the squared memory block. This module provides the symbol timing estimation.

## 5. Experimental Results

The synchronization algorithm proposed previously has been implemented in a KC705 platform [37] by Xilinx Inc. (San Jose, CA, USA), which is based on a Kyntex XC7K325T FPGA [38]. Regarding the resource consumption, Table 1 shows the figures for the main logic elements available in the device. Generally speaking, the reduced utilization percentage can be observed. Furthermore, Table 2 details this resource consumption for every module involved in the proposed design. In this case, the most of the required resources are dedicated to the correlation module, where it is necessary to implement a FFT block in charge of parallelizing the input data. This minimizes the latency cycles required to obtain every output sample.

**Table 1:** Resource consumption of the synchronization algorithm implementation in a Xilinx XC7K325T FPGA.

	Proposed Design	Utilization Percentage
**Flip-Flops**	27,046	6%
**RAMB**	68	7%
**LUTs**	21,207	10%
**DSP48E1**	236	28%

**Table 2:** Detailed resource consumption for the main modules of the design in a Xilinx XC7K325T FPGA.

	Flip-Flops	RAMB	LUTs	DSP48E1
**Global Design**	27,046	68	21,207	236
**Correlation Module**	26,388 (97.57%)	64 (94.12%)	20,587 (97.08%)	220 (93.22%)
**Squaring Module**	12 (0.04%)	0 (0.00%)	10 (0.05%)	16 (6.78%)
**Maximum Detection**	325 (1.20%)	0 (0.00%)	343 (1.62%)	0 (0.00%)
**Thresholding Module**	14 (0.05%)	0 (0.00%)	177 (0.65%)	0 (0.00%)

The proposed design works at a clock frequency of *f_CLK_* = 50 MHz, thus requiring 1024 input data every 64 clock cycles (16 18-bit samples every clock cycle), with a latency of 580 cycles. In this case, it is assumed that the sampling frequency at the receiver is *f_S_* = 50 Msps in order to fill up a buffer with a length 2(*M* + *L_cp_*), for *M* = 512 subchannels and cyclic prefix length *L_cp_* = 400. In a parallel way, the synchronization delay is estimated for the previous buffer, providing a final estimation after a latency of 580 cycles for a clock frequency of *f_CLK_* = 50 MHz. This implies a long enough interval in order to synchronize the receiver and not to discard input samples at the reception. Since the latency may still increase without any drawback for the system operation, the resource consumption in the correlation module could be minimized by parallelizing even more the data input.

Another aspect that has been analyzed is the quantization error due the fixed-point representation used in FPGA-based designs. This study has been focused on the two modules where the most intensive computation is carried out: the correlation and the squaring modules. Note that the maximum detection module and the thresholding module imply comparisons and assignments, but not arithmetical operations where quantization errors could have influence on them. Table 3 shows the maximum relative error and the averaged relative error for the input signal *r*[*n*], for the correlation output *x*[*n*], and for the squared output *y*[*n*], obtained by performing 1000 simulations with different channel models according to [25,36], for a SNR = 10 dB. Due to the fact that all the fractional bits are discarded at the output *y*[*n*] of the squaring module, quantization errors become more relevant. Nevertheless, this signal *y*[*n*] is only used further to detect maximum values in correlation, so the global performance of the system is not degraded by this decision.

**Table 3:** Relative quantization errors for the main signals in the proposed design.

	Maximum Relative Error	Averaged Relative Error
**Input *r*[*n*], Q(18.8)**	0.055%	0.052%
**Correlation output *x*[*n*], Q(18.8)**	0.132%	0.117%
**Squared output *y*[*n*], Q(18.0)**	7.98%	4.72%

Finally, to validate the proposed synchronization algorithm and its FPGA-based implementation, some simulation results have been performed. The study has been carried out for two different PLC channel model parameters, where signal-noise ratios (SNR) from −5 dB to 30 dB, in steps of 5 dB, have been configured. For every configuration, 1000 simulations have been performed. Regarding the PLC channel model, it is based on the Tonello PLC channel model [36], where two parameter configurations have been considered: the channel B with more favorable parameters [36] and channel A, more complex and obtained from [25]. Table 4 describes the main parameters for both models.

**Table 4:** Parameters for the PLC channel model considered here [25,36].

	Channel B	Channel A
Maximum length	300 m	800 m
Attenuation dependent of the frequency *a_0_*	10^−5^	0.3 × 10^−2^
Attenuation dependent of the frequency *a_1_*	10^−9^	4 × 10^−2^
Poisson arrival time intensity	0.667 m^−1^	0.2 m^−1^
Channel length	4 µs	5.56 µs
Stop frequency	31.25 MHz	31.25 MHz

**Figure 10:**
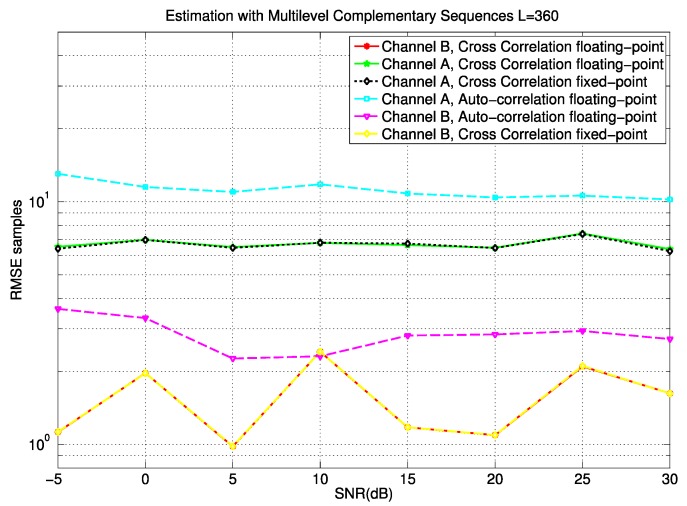
RMSE in the synchronization delay estimation for both channels, and for a floating- and a fixed-point representation and the auto-correlation method.

Furthermore, four different types of noise have been evaluated: synchronous impulsive noise, asynchronous impulsive noise, background noise, and narrowband noise; whose power levels are defined in [20]. Figure 10 plots the Root Mean Squared Error (RMSE) for the synchronization delay estimation with respect to the SNR level, taking into account both channel models A and B. It can be observed that the difference between the floating-point algorithm and the fixed-point version implemented in the FPGA device is negligible. Furthermore, it can be verified how the RMSE for the model A is higher than the one for the model B, due to the complexity of this type of PLC channel. In order to compare the proposal with other approaches, Figure 10 also shows the RMSE obtained for the auto-correlation method [22]. Note that its RMSE values are higher than those achieved by the proposed algorithm for both channel models A and B, so the proposed synchronization algorithm improves the performance provided by auto-correlation methods. For every configuration, the RMSE value presents a fluctuation below one sample, which is due to the statistical estimation carried out (1000 simulations for every SNR value). The figures have been obtained by means of multilevel complementary sequences with a length *L* = 360 bits.

**Figure 11:**
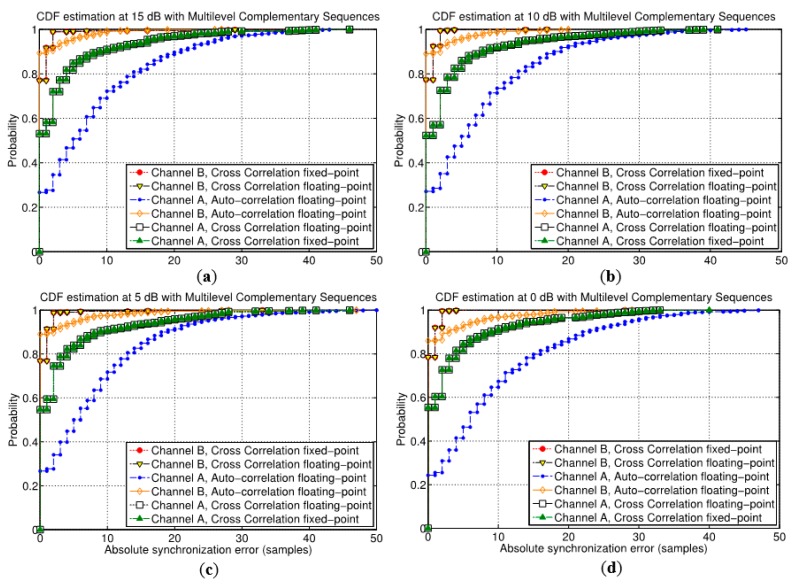
Cumulative distribution function (CDF) of the absolute error in the synchronization delay estimation with different SNR values, (**a**) 15 dB, (**b**) 10 dB, (**c**) 5 dB, (**d**) 0 dB, for both models A and B, for the auto-correlation method and for the floating- and fixed-point versions of the proposed algorithm.

On the other hand, Figure 11 shows the cumulative distribution function (CDF) of the absolute error in the synchronization delay estimation for different SNR values and using multilevel complementary sequences. Note that the absolute error is considered as the absolute difference in samples between the estimated synchronization delay and the real delay. The CDF is plotted for both channel models A and B, and not only for the proposed algorithm based on cross correlation but also for the auto-correlation approach. Furthermore, the proposed synchronization algorithm based on cross correlation is evaluated in its corresponding floating- and fixed-point representations. In general terms, the proposed algorithm achieves a better performance than the auto-correlation one, especially for the more complex channel model A regardless the SNR value. In the case of the simpler channel model B, the auto-correlation approach can provide a higher rate of ideal estimation of the synchronization delay, but in case of not achieving a perfect estimation, the errors in the delay estimation are greater than the proposed cross-correlation algorithm. Also, the FPGA-based architecture proposed for the fixed-point implementation provides negligible differences with the floating-point version (note that both plots are almost overlapped in Figure 11). Taking into account the analyzed SNR values, it can be observed the immunity to noise of the proposal, since the performance is not degraded as the noise level increases.

## 6. Conclusions

The PLC channel shows several particularities, such as severe multipath or non-Gaussian noises in the mains, which prevent the direct use of traditional synchronization techniques in wireless communication systems. In this paper a symbol timing estimator based on cross-correlation is proposed, as well as its efficient hardware implementation in a FPGA device, where the use of multilevel complementary sequences or Zadoff-Chu sequences as pilot symbols provides robustness to the synchronization algorithm. Although the Cyclic Prefix is not needed theoretically for broadband PLC with Wavelet-OFDM as medium access technique, it allows the decrease of the required resource consumption at expense of reducing bandwidth efficiency.

To minimize the number of multipliers involved in the correlation stage, the DFT block has been reused in order to avoid an additional IDFT block, and a pipelined architecture has been designed to minimize the system latency and to maximize data throughput, whereas using the minimum number of hardware resources in the FPGA. The quantization errors in the proposed symbol timing algorithm have been also compared between a fixed-point representation and a float-point representation, showing negligible values in the worst case. The proposed synchronization algorithm is capable of estimating the first arriving path without any errors in the 90% for the Tonello PLC channel model B.
